# ﻿Digitization of the historical Herbarium of Michele Guadagno at Pisa (PI-GUAD)

**DOI:** 10.3897/phytokeys.234.109464

**Published:** 2023-10-12

**Authors:** Francesco Roma-Marzio, Simonetta Maccioni, David Dolci, Giovanni Astuti, Nicoletta Magrini, Federica Pierotti, Roberta Vangelisti, Lucia Amadei, Lorenzo Peruzzi

**Affiliations:** 1 Orto e Museo Botanico, Sistema Museale d’Ateneo, Università di Pisa, via Ghini 13, 56126 Pisa, Italy Università di Pisa Pisa Italy; 2 PLANTSEED Lab, Dipartimento di Biologia, Università di Pisa, via Derna 1, 56126 Pisa, Italy Università di Pisa Pisa Italy

**Keywords:** herbaria, historical botanical collection, history of botany, museology, specimen metadata, taxonomy

## Abstract

The herbarium digitization process is an essential first step in transforming the vast amount of data associated with a physical specimen into flexible digital data formats. In this framework, the Herbarium of the University of Pisa (international code PI), at the end of 2018 started a process of digitization focusing on one of its most relevant collections: the Herbarium of Michele Guadagno (1878–1930). This scholar studied flora and vegetation of different areas of southern Italy, building a large herbarium including specimens collected by himself, plus many specimens obtained through exchanges with Italian and foreign botanists. The Herbarium is composed by 547 packages of vascular plants. Metadata were entered into the online database Virtual Herbaria JACQ and mirrored into a personalized virtual Herbarium of the Botanic Museum. After the completion of the digitization process, the number of sheets preserved in the Herbarium amounts to 44,345. Besides Guadagno, who collected 42% of his specimens, a further 1,102 collectors are represented. Most specimens were collected in Europe (91%), but all the continents are represented. As expected, Italy is the most represented country (59%), followed by France, Spain, Germany, and Greece. The specimens cover a time span of 99 years, from 1830 to 1929, whereas the specimens collected by Guadagno range between 1889 and 1928. Furthermore, we traced 134 herbarium sheets associated with documents, among which 75 drawings handmade by Guadagno, 34 letters from various corresponding authors, 16 copies of publications, and 14 copies of published iconographies.

## ﻿Introduction

Herbarium specimens, as all the other natural history collections, are verifiable records of the presence of a species in a defined place at a specific time (Borsch et al. 2020). Currently, around 396 million specimens are kept in more than 3,000 active herbaria and can be considered as the basic documentation of all formally described plant species (Thiers 2021; Heberling 2022).

The inestimable value of herbaria is widely recognised in the fields of taxonomy, systematics, and biogeography and, in the last decades, they turned out to be greatly useful for studying ecological shifts (Lang et al. 2019; Garretson and Forkner 2021; Davis 2023; Jaroszynska et al. 2023). Indeed, herbarium studies should not be limited to the most recent specimens, but also to historical ones (e.g., Stefanaki et al. 2019), that have proven to be fundamental in several biological disciplines, from the most traditionally prone to the use of herbaria (Funk 2018) to the most recent approaches dealing with climate change biology (Heberling 2022), biodiversity (Nelson and Ellis 2018), phenology (Park et al. 2023), nature conservation (Nualart et al. 2017), biological invasions (Martin et al. 2014), and phylogenomics (Beck and Semple 2015). Also, herbaria are the repositories of nomenclatural types, and hold useful data for studies on endangered species or represent a documentation of taxa that have gone extinct in historical times (Lavoie 2013). Actually, herbaria may be the only source for the resurrection of extinct species, or de-extinction, because they may preserve viable diaspores (Albani Rocchetti et al. 2022a, b).

Given the high scientific, historical, and cultural value of herbarium collections, in the last 20 years many efforts have been made towards their digitization, and to make them available to the scientific community and beyond (Tulig et al. 2012). The digitization process – i.e. the capture of images and metadata from specimen labels – is an essential first step in transforming this vast amount of data associated with a physical specimen into flexible digital data formats that are accessible, usable, and useful (Hedrick et al. 2020), since it allows information to be summarised, categorised and manipulated, in order to retrieve specific information, otherwise hidden in an overwhelming pile of specimens. Particularly, historical specimens are often accompanied by fragmentary label information, so that providing a link to correct metadata is crucial to make them accessible for researchers worldwide (Reiner-Drehwald et al. 2022).

However, digitization activities require huge efforts in terms of equipment, personnel training and, consequently, time and money. Although the digitization rates increase in parallel with the experience of technical staff, databasing is the most time-expensive step with effort variable according to the details of metadata to capture, which can vary from a complete databasing including georeferentiation to a minimal subset of label data referred as skeletal (Powell et al. 2021).

In this framework, the Herbarium of the University of Pisa [PI is the international code according to Thiers (2020, and onwards)], started a process of digitization in November 2017. The PI Herbarium, currently preserving more than 350,000 specimens from all over the world, was started by Gaetano Savi (1769–1844), director of the Pisa Botanic Garden from 1814 to 1842. His first collection dates back to the end of the XVIII century, when he collected the first specimens during botanic trips with Giorgio Santi (1746–1822). Furthermore, thanks to the friendship with Ottaviano Targioni Tozzetti (1755–1826), he had access to the collection of Pier Antonio Micheli (1679–1737) and he had also the opportunity to bring to Pisa some of these specimens, which are the oldest specimens currently preserved in the Herbarium. In addition to the general Herbarium, other separate collections are preserved in PI, such as the Herbarium of Michele Guadagno (1878–1930). After his death, in 1939 this Herbarium (PI-GUAD hereafter) was sold by his relatives to PI. In December of the same year, Alberto Chiarugi (1901–1960), the director of the Botanical Institute in Pisa, with the help of his son, personally carried the Herbarium to Pisa, and counted 36,648 specimens only considering those already intercalated (Chiarugi 1950). It has been hence considered one of the richest private herbaria ever recorded in Italy.

Michele Guadagno was born in Naples (Campania, southern Italy) on October 16^th^ 1878 and showed from an early age a special attitude for natural sciences and particularly for botany. In 1905 he graduated as a civil engineer in Naples and then he worked as an engineer at the Municipality of Naples (Trotter 1930; D’Erasmo 1931; Andolfato et al. 2002). During his life, he studied the flora and vegetation of different areas of Campania, which led to seventeen papers between 1909 and 1932, including a monograph on the vegetation of the Sorrento Peninsula and a floristic study of Capri Island, posthumously published by his friend Augusto Béguinot (1875–1940). Unfortunately, the former monograph is incompletely published, as only three out of the six parts planned by the author were published, while the fourth part was only partially published. The complete botanic bibliography of M. Guadagno is available on the online Suppl. materal 1: table S1. Alongside the scientific activities resulted in publications, he built a large herbarium including specimens collected by himself mainly in central and southern Italy, plus many specimens obtained through exchanges with Italian and foreign botanists. As a result of these exchanges, specimens collected by Guadagno can be also found in numerous Herbaria like MA, L, SPA, P (a list of all the Herbaria where we were able to track specimens collected by Guadagno is available in Suppl. materal 1: table S2). PI-GUAD is accompanied by an index in three volumes compiled by Guadagno, and containing the list of taxa and the corresponding number of specimens annotated into two columns with the heading “G” and “D”, meaning those collected by Guadagno and those donated by other botanists, respectively. Also, Guadagno left a card index, now housed at the Herbarium of Naples [code NAP according to Thiers (2020, and onwards)], containing meticulous taxonomic, nomenclatural and biogeographical annotations about the species he encountered during his more than 200 field trips (Fontanella 2008).

PI-GUAD also preserves original material for taxa described by other botanists (accepted names in square brackets), like Drabalongirostravar.guadagnoi O.E.Schulz (1927; see also Mazzola et al. 2014), *Violasplendida* W.Becker [≡ Violaaethnensis(Ging. & DC.)Stroblsubsp.splendida (W.Becker) Merxm. & Lippert] (Becker 1902; Portal to the Flora of Italy 2023), Hieraciumsartorianumvar.lucanicum Arv.-Touv. [= HieraciumhypochoeroidesS.Gibsonsubsp.lucanicum (Arv.-Touv.) Di Grist., Gottschl. & Raimondo] (Guadagno 1909; Di Gristina et al. 2015), Centaurea×cavarae Guadagno ex Del Guacchio, Cennamo & P.Caputo (Del Guacchio et al. 2020a), and it has been an important source of data for floristic and taxonomic studies (Ricciardi 1998; Andolfato et al. 2002; Del Guacchio and Caputo 2008; Gottschlich 2009; Peruzzi et al. 2013, 2015a, 2019a; Roma-Marzio et al. 2015, 2017a, 2018; Conti et al. 2019; Del Guacchio et al. 2020b; Gargano et al. 2023).

For all these reasons, at the end of 2018, the Botanic Museum of the University of Pisa started a project in collaboration with the Department of Biology, focused on the digitization of all the vascular plant specimens of the Herbarium of Michele Guadagno. The aim of this paper is to illustrate the process of digitization of the Herbarium Guadagno, which made the collection freely accessible to botanists. The digitization process allowed to extract and summarise information about collectors, localities, collection dates, and taxonomy of the specimens, including nomenclatural types.

## ﻿Materials and methods

**Figure 1.**
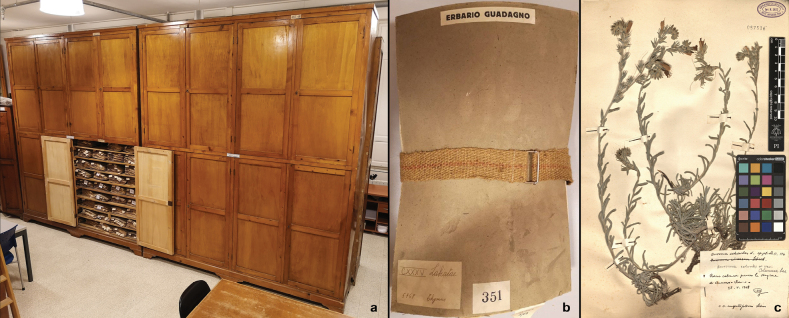
Two of the four cabinets where is preserved the Herbarium of Michele Guadagno (PI-GUAD) at the Botanic Museum of the University of Pisa (**a**), one of the 547 packages of the collection (**b**), and an example of a specimen collected by Guadagno (**c**).

PI-GUAD, stored inside four cabinets of 310 × 270 × 58 cm and another small cabinet of 110 × 68 × 50, is composed by 547 packages of vascular plants, of which 501 organised according to the systematic criterion introduced by Durand (1888), and other 39 organised according to collection localities or correspondents of Michele Guadagno (Fig. 1). Concerning the seven packages of pteridophytes, they were reassembled by extracting the specimens that were previously interspersed in the general Herbarium of Pisa. PI-GUAD also includes specimens of non-vascular plants, algae, fungi, and lichens, not considered in this study.

Prior to the acquisition of the images, all the specimens lacking a scientific name were at least identified at the genus level. Then, a unique identification number (ID hereafter) was stamped on each specimen.

The digitization procedure followed two distinct steps: 1) acquisition of high-resolution digital images and 2) label data (metadata) acquisition.

Concerning the image acquisition, each specimen was scanned using a Bookeye 4 Professional planetary scanner producing a 600 dpi and 24-bit color depth image in .tiff format. Each scan was accompanied by a metric and colorimetric reference. After the acquisition, each image was renamed using the ID assigned to the specimen and uploaded on a web server of the University of Pisa. To produce a backup copy, all the images were also converted in .jpeg format and stored in physical hard disks preserved at the Botanic Museum.

Metadata were entered into the online database Virtual Herbaria JACQ (http://www.jacq.org/), a continuously developing consortium of virtual herbaria located in Vienna (Bräuchler et al. 2021). Today JACQ is used by 53 institutions in 18 countries worldwide, mainly in Europe, with a total of approximately 1,400,000 records covering the entire globe and constituting an important source for collection data portals such as GBIF and BioCASe (Borsch et al. 2020). One of the strengths of this free project is the presence of a query system which allows searching simultaneously all the Herbaria that are partners of the project. The Virtual Herbarium JACQ allows for structured registration of metadata, georeferencing of each specimen, and automatic linking of these data to digital images. In addition, it is possible to download a .csv file containing all the metadata of the recorded specimens, as well as a .kml file containing the geographical data, useful for GIS-based analyses. By means of the .csv file produced by JACQ we also set up a personalized Virtual Herbarium of the Botanic Museum (https://erbario.unipi.it), that mirrors the data inserted into JACQ.

To detect original material and type specimens preserved in the Herbarium, we analysed the literature that directly cites specimens from PI-GUAD, and investigated the protologue of all those taxa whose author(s) were contemporaries of Guadagno or were the collector of the specimens, or when the labels bear annotations like “*sp. nov*.”, “*var. nov*.” or similar. Furthermore, to trace duplicates of types, we also analysed literature with massive data about typification of taxa described between the end of XIX and the first decades of XX century, or described by authors which were collectors in the Herbarium Guadagno (Bartolucci et al. 2013; Peruzzi et al. 2015b; 2019b; Galasso et al. 2018a; Vogt et al. 2018; Raptis et al. 2019; Del Guacchio et al. 2020a, 2020b; Reich et al. 2021). All the type specimens detected were also uploaded in the JSTOR Global Plants, the world’s largest database of digitized plant specimens (JSTOR Global Plants 2019).

At the end of the digitization process, each specimen was marked with a stamp (“D”) to indicate that the digitization process was completed.

Concerning the georeferentiation, coordinates were inferred from the geographical information reported on the label, which range from very precise indications of the locality to very generic indications of a wide geographic area. For collectors, we referred to the Harvard University Herbaria Index of Botanists (https://kiki.huh.harvard.edu/databases/botanist_index.html) as a guide for handwritings hard to decipher. When possible, we inferred the collectors based on the handwriting’s recognition if not explicitly reported (e.g., the many specimens collected by Guadagno).

For the analysis of the plant families, we updated the default taxonomic circumscription used by JACQ according to PPG I (2016) for ferns and fern allies, to Christenhusz et al. (2011) for gymnosperms, and to APG IV (2016) for angiosperms.

Since many specimens are represented or accompanied by drawings, illustrations, manuscripts, correspondences, publications, and other documents, these specimens were marked as “.doc” in the “annotations” field of the database, to facilitate their search.

All the statistical analyses and graphs were carried out by means of PAST version 4.11 (Hammer et al. 2001; Hammer 2022), whereas QGIS 3.10 software was used to draw all the maps.

## ﻿Results

The number of sheets preserved in PI-GUAD amounts to 44,345, to which a further 65 not digitized sheets, lacking label, should be added. The number of specimens amounts to 41,314. The reason for this discrepancy lies in the admixture of specimens in some sheets, and in the occurrence of specimens composed by two or more sheets. Among the digitized specimens there are also 16 sheets bearing 66 labels of specimens destroyed during the flood of Arno River in 1944.

From a taxonomic point of view, the Herbarium includes pteridophytes (833 specimens corresponding to 2% of the collection), gymnosperms (177; 0.4%), non-eudicot dicots (114; 0.3%), monocots (6,730; 16.3%), and eudicots (33,457; 81%). Totally, 255 families and 2,380 names at the genus level (including names currently considered as synonyms) can be found in PI-GUAD. The ten most represented families are Asteraceae (5,970; 14.5%), Fabaceae (3,981; 9.6%), Poaceae (3,347; 8.1%), Lamiaceae (2,260; 5.5%), Caryophyllaceae (2,070; 5%), Brassicaceae (1,794; 4.3%), Apiaceae (1,524; 3.7%), Rosaceae (1,330; 3.2%); Ranunculaceae (1,179; 2.9%), Plantaginaceae (1,171; 2.8%). The most represented names at genus level are: *Hieracium* (701; 1.7%), *Carex* (677; 1.6%), *Trifolium* (668; 1.6%), *Centaurea* (646; 1.6%), *Silene* (559; 1.4%), *Vicia* (534; 1.3%), *Ranunculus* (518; 1.3%), *Galium* (423; 1.0%), *Viola* (422; 1%), *Euphorbia* (404; 1%). A complete list of all families, genera and relative number of specimens are available in the online Suppl. material 1: table S3.

**Figure 2.**
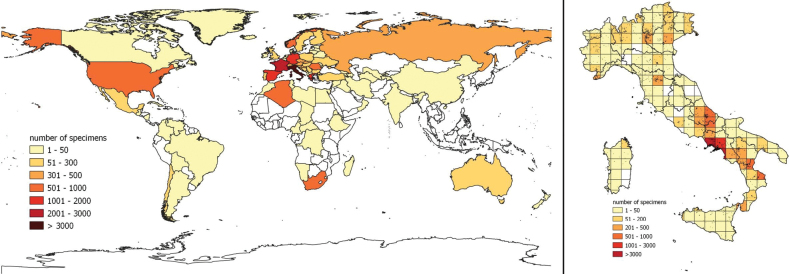
Global geographic coverage of the specimens preserved in the herbarium of Michele Guadagno (PI-GUAD) at the Botanic Museum of the University of Pisa (**a**), and focus on specimens collected in Italy (**b**). The number of specimens in Italy is calculated on a grid of 50×50 km.

**Figure 3.**
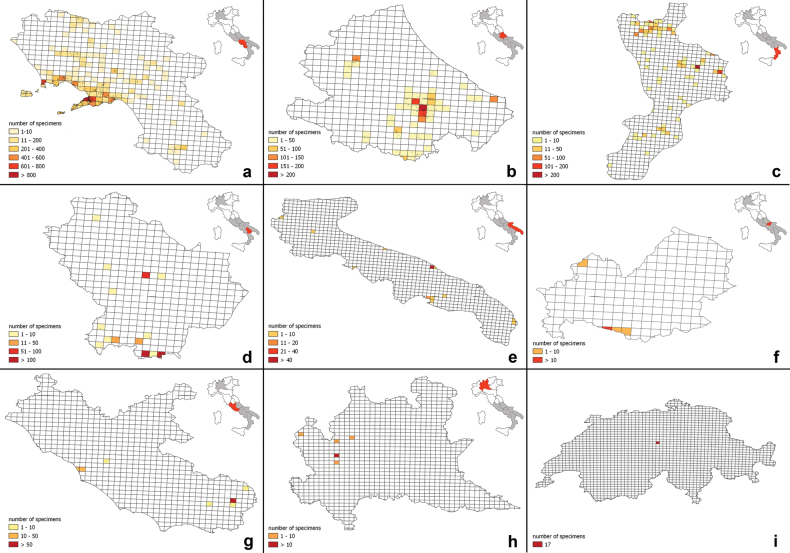
Geographic coverage of the specimens preserved in the Herbarium of Michele Guadagno (PI-GUAD) at the Botanic Museum of the University of Pisa and collected by Michele Guadagno in Italy (**a** Campania **b** Abruzzo **c** Calabria **d** Basilicata **e** Puglia **f** Molise **g** Lazio **h** Lombardia) and Switzerland (**i**). The number of specimens is calculated on a grid of 5×5 km.

From the geographical point of view, although most of the specimens were collected in Europe (91.8%), there are specimens from all the other continents: Africa (4.3%), Asia (1.8%), North America (1.8%), South America (0.2%), and Oceania (0.7%). Overall, there are 90 countries in which at least one specimen was collected: Italy is, of course, the most frequent (24,372, 59.1%), followed by France (2941; 7.1%), Spain (1,618; 3.9%), Germany (1,299; 3.2%), and Greece (1,234; 3%) (Fig. 2a). A complete list of all geographic data is available in the online Suppl. material 1: table S4. As concerns the Italian regions, Campania is the most represented (13,027; 54.2%), followed by Abruzzo (2,628; 10.9%), Calabria (1,800; 7.5%), Veneto (1,042; 4.3%), and Toscana (820; 3.4%) (Fig. 2b). A focus on the specimens collected by Guadagno shows that he collected plants mainly in Campania (12,482), followed by Abruzzo (2,274), Calabria (1,611), Basilicata (365), Lazio (141), Puglia (83), Molise (36), Lombardia (27) and, out of Italy, in Switzerland (17) (Fig. 3).

**Figure 4.**
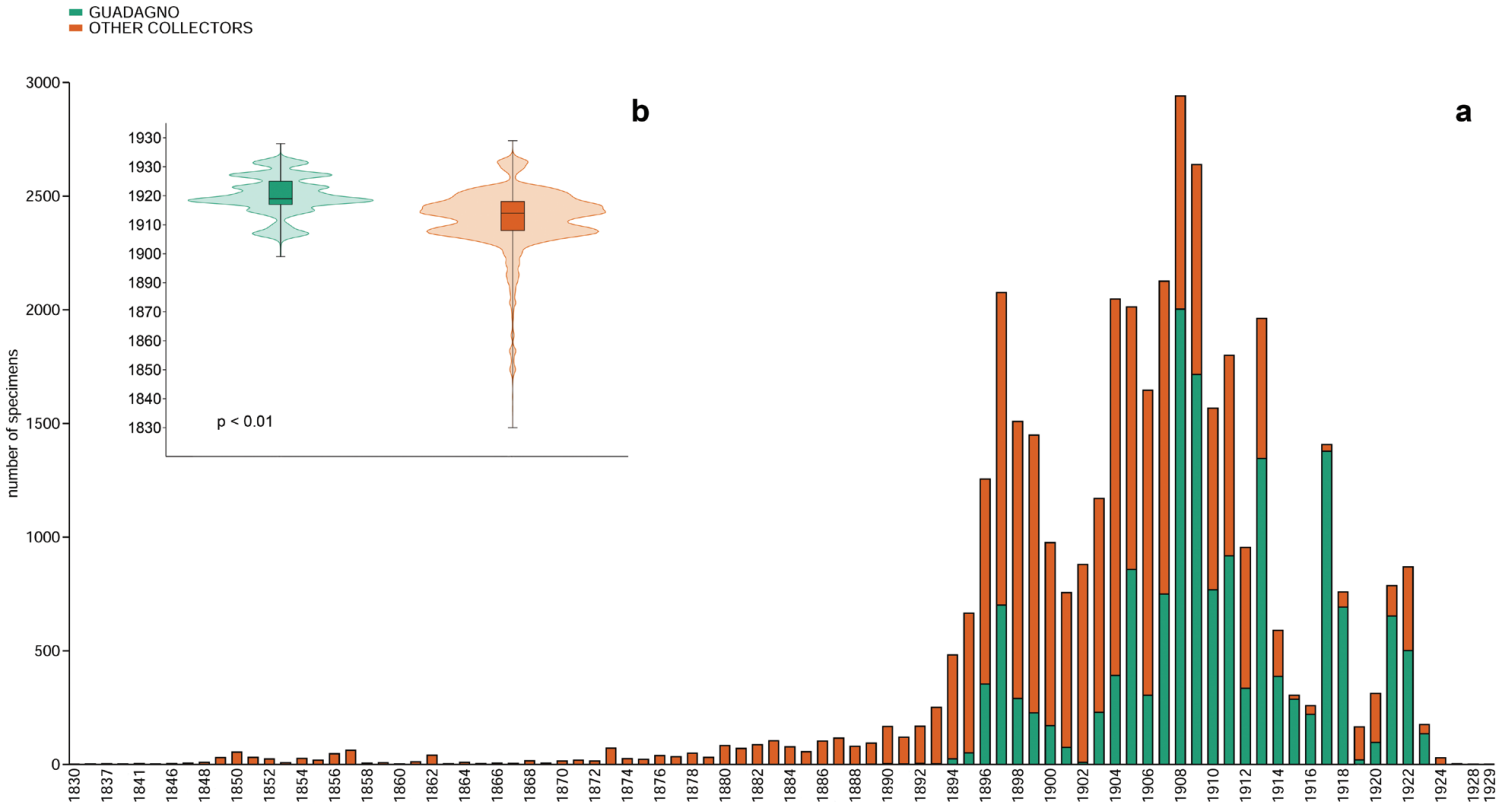
Histograms showing temporal coverage of specimens preserved in the Herbarium of Michele Guadagno (PI-GUAD) at the Botanic Museum of the University of Pisa (**a**). Boxplots show the differences in the collection year among specimens collected by Guadagno and other collectors (**b**).

All the digitized specimens cover a time span of 99 years, from 1830 (48 years before the birth of Guadagno) to 1929 (Fig. 4) (see discussion for further details), whereas the specimens collected by Guadagno range between 1889 and 1928. The variation of the collection effort through years is significantly different between Guadagno (median year 1909; range 39) and the other collectors (1904; 99) (Mann-Whitney test p < 0.01).

Besides Guadagno, who collected 42% of the specimens, another 1,103 collectors are present. Nonetheless, 319 specimens (1%) do not report any collector, while for other 90 specimens (0.2%) we were unable to decipher the collector’s name. Among the other collectors, the most frequent are A. Noblet (France) with 779 specimens (1.9%), C. Marchesetti (Italy, Slovenia, and Croatia) with 680 specimens (1.6%), W. Behrendsen (Germany, Bosnia and Herzegovina, Poland, Croatia, Montenegro) with 679 specimens (1.6%), C. Bicknell (Italy and France) with 632 specimens (1.5%), J. M. Wood (South Africa) with 561 specimens (1.4%), S. Sommier (Italy) with 539 specimens (1.3%), and G. Rigo (Italy) with 514 specimens (1.2%). Considering only the specimens collected in Italy, there are 217 collectors besides Guadagno, among them the most frequent are: G. Rigo (512 specimens), S. Sommier (497), C. Bicknell (391), A. Fiori (374), A. Béguinot (369), O. Gavioli (346), A. Mazza (336), L. Grande (324), C. C. Lacaita (305), and R. Pampanini (303). A complete list of all collectors and relative data is available in the online Suppl. material 1: table S5.

PI-GUAD hosts 4,291 specimens linked to 133 different series of exsiccata; among them the most represented are “Flora Italica Exsiccata series I” by A. Fiori, A. Béguinot and R. Pampanini (1,072 digitized specimens, 28 of which collected by Guadagno himself) and “series II” by A. Fiori and A. Béguinot (1,154, 30 of which collected by Guadagno), “Herbarium Graecum Normale” by T. von De Heldreich (258), “Plantes d’Espagne” by F. Sennen (277), “Plantes d’Espagne” by Elisée Réverchon (176), and “Herbarium Normale” by I. Dörfler (85). A complete list of all the series of exsiccata is available in the online Suppl. material 1: table S6.

We traced 134 sheets bearing plants associated with documents or only documents. In particular, we found 77 drawings made by Guadagno or by his corresponding authors, 16 copies of publications and 14 copies of published iconographies. All these sheets can be found in JACQ database or in the Virtual Herbarium of the Botanic Museum by typing “_doc” in the field “annotation”.

After the digitization process, we traced 44 specimens that can be considered original material for several names not yet typified, and another 62 type specimens, of which one is a holotype, 15 are isotypes, four are lectotypes, 16 are isolectotypes, 25 are syntypes, and one is an isoepitype.

## ﻿Discussion

The digitization of PI-GUAD revealed much information hidden in the cabinets, confirming the importance of this procedure to study large natural collections (James et al. 2018).

Considering only the first 501 packages of the Herbarium, which include the material organized according to a systematic criterion, we surveyed 40,748 herbarium sheets, 4,100 more than those previously counted by Chiarugi (1950).

The number of plant families represented in PI-GUAD covers 58% of the 452 families of vascular plants of the world, and the four most represented families in the Herbarium (Asteraceae, Fabaceae, Poaceae, and Lamiaceae) correspond, in this precise order, to the four most represented families at global scale (Christenhusz and Byng 2016).

Besides Italy, which is the country most represented, the geographic data at European scale are considerable, providing useful information for research on plant diversity in this continent, as already emerged by other studies using data from PI-GUAD (Janković et al. 2019). Focusing on Italy, the results of the digitization confirmed that most of the specimens were collected by Guadagno and his correspondents in the southern regions, and particularly in Campania (Amadei et al. 2013; Roma-Marzio et al. 2017b). This precious source of data is confirmed also by several publications of floristic records based on specimens preserved in PI-GUAD (Badino and Peruzzi 2009; Del Guacchio 2010; Bartolucci and Santangelo 2015), that contributed to compile the updated checklist of the vascular floras native and alien to Italy (Bartolucci et al. 2018; Galasso et al. 2018b).

PI-GUAD also hosts specimens of taxa that are currently extinct or, according to IUCN criteria (IUCN 2022), evaluated Endangered or Critically Endangered in Italy (Rossi et al. 2013; 2020). An emblematic example is represented by the single specimens of *Ipomoeaimperati* (Vahl) Griseb. (under the name *Convolvulusimperati* Vahl; PI nr. 057590), collected by Giulio Avellino on the Ischia Island and donated by Giuseppe Antonio Pasquale (1820–1893) to Guadagno. This species was considered extinct in Campania until the recent rediscovery by Bartolucci et al. (2022) near Naples. The specimen preserved in the Herbarium therefore is a case of useful source of genetic material or propagules for driving practical in situ and ex situ conservation actions (Albani Rocchetti et al. 2021), or for effective recovery of genetic variation and structure of lost population in wild, as already investigated for this species (Cennamo et al. 2013). Another case is represented by the specimen of *Eokochiasaxicola* (Guss.) Freitag & G.Kadereit (under the name *Kochiasaxicola* Guss.; PI nr. 038612) collected by Guadagno on the Ischia Island. This species, currently evaluated as Endangered in Italy (Rossi et al. 2013), is only known for three populations, two located in Capri Island and Palinuro in Campania, one in Strombolicchio islet in the Aeolian Archipelago in Sicily, whereas the population from Ischia is currently extinct (Strumia et al. 2021).

As concerns the collectors, although 42% of the specimens were collected by Guadagno, the high number of other collectors and the different series of exsiccata testify to the extensive exchanges among Guadagno and other botanists from all over the world (Trotter 1930; D’Erasmo 1931). This is also testified by the 34 letters we found intermixed with specimens. Another evidence of Guadagno relationships with other prominent botanists throughout Europe is represented by the revisions of many specimens sent to specialists of certain genera or groups of plants, such as Jean-Maurice Casimir Arvet-Touvet (1841–1913) for *Hieracium*, Émile Burnat (1828–1920) for *Rosa*, Eduard Hackel (1850–1926) for Poaceae, Hermann Christ (1833–1933) for pteridophytes (see also Marchetti 2021).

The first specimen collected by Guadagno is a plant of *Fallopiaconvolvulus* (L.) Á.Löve (under the name *Polygonumconvolvulus* L.) found in Naples, in July 1889 when he was only eleven years old (PI nr. 024486), the label that reports “*Polvica (Capodimonte) (Prop. Perretti)*” probably refers to an ancient farmhouse near Chiaiano (E. Del Guacchio pers. comm.) property of the family Perretti, surname of the mother of Guadagno Rosa Perretti (D’Erasmo 1931). The last specimen collected by Guadagno (PI nr. 033129) is a plant of *Hyparrheniahirta* (L.) Stapf, from Mt. Barbaro (Campania) in 1928, two years before his death. The span between the dates of these two specimens covers 75% of the life span of Michele Guadagno, testifying that his passion for natural science maintained until the end of his days (Trotter 1930). The specimens collected by the other collectors cover a wider temporal range and are generally older with respect to those collected by Guadagno. He accumulated a fair number of specimens and had the possibility to exchange some of them with older botanists that started their activities earlier.

The analysis of the collection data revealed some biases in the collection activities made by Guadagno, particularly between 1919 and 1920, when the collections were reduced with respect to other periods, probably because this was the busiest time for Guadagno as an engineer (Trotter 1930). Another significant reduction can be seen between 1915 and 1918 considering the specimens collected by his correspondents, probably due to World War I, a trend also highlighted in other Herbaria (Daru et al. 2017).

Among the specimens collected before the birth of Michele Guadagno, those collected by three notorious Italian botanists like Giovanni Gussone (1787–1866), Filippo Parlatore (1816–1877), and Antonio Bertoloni (1775–1868) are of particular interest. There are seven specimens collected by Gussone, almost all of them with labels handwritten by Loreto Grande, a friend of Guadagno and curator of the Naples Herbarium between 1921 and 1942. In these years, Grande sent many specimens to various botanists for revision (Santangelo et al. 1995) including Guadagno, who was probably unable to return these specimens to NAP because of his sudden death. This hypothesis is also supported by considering that in those years the director of the Botanical Garden of Naples was Fridiano Cavara (1857–1929), a point of reference and friend of both Guadagno and Grande. Two of the specimens collected by Gussone refer to taxa that he described (*Asperulanitens* Guss. PI nr. 061806, and *Sonchusnymanii* Tineo & Guss. PI nr. 035943) and collected in their type localities (Gussone 1843; Del Guacchio and Caputo 2005), whereas the specimen of *Alyssumhispidum* Loscos & J.Pardo (PI nr. 034437) was collected during his journey in Spain (Trotter 1948). In the case of Bertoloni, we traced one specimen (PI nr. 052728) collected in the Botanical Garden of Bologna, where Bertoloni served as Praefectus from 1817 to 1869. It is not clear why this specimen ended up in the hands of Guadagno, but we can hypothesize that Federico Delpino (1833–1905), who was the Praefectus at Bologna’s Garden from 1884 to 1894 (Mossetti 2021), and then moved to the Botanical Garden of Naples until 1905 (Catalano 1958), might have acted as the intermediary of this passage. Alternatively, we may infer that this specimen was sent by Bertoloni to Tenore or Gussone (as many others in NAP), and only later came into the hands of Guadagno, who was unable to return them because of his sudden death. The presence of specimens like these may be difficult to trace by researchers. Therefore, the digitization activities, including those concerning private or separate collections, can be very useful to locate specimens otherwise possibly considered as lost.

Among the 62 type specimens traced in the Herbarium, there are 15 types of taxa currently accepted at the original or different rank: an isotype of AdonisflammeaJacq.subsp.cortiana C.H.Steinb. (PI nr. 015061, Steinberg 1971); a lectotype of HieraciumsartorianumBoiss. & Heldr.var.lucanicum Arv.-Touv. (PI nr. 019816, Di Gristina et al. 2015), isolectotypes of *Dianthustarentinus* Lacaita (PI nr. 040642, Bacchetta and al. 2010), *Globularianeapolitana* O.Schwarz (PI nr. 013730, Del Guacchio et al. 2020a), *Muscarilongifolium* Rigo (PI nr. 025613, Frattini et al. 1996; Galasso et al. 2018a), *Nepetaheldreichii* Halácsy (PI nr. 039187, Baden 1987; Reich et al. 2021), PolygalaflavescensDC.var.maremmana Fiori (PI nr. 030515, Arrigoni, 2014), PolygalaalpestrisRchb.var.valdarnensis Fiori (PI nr. 051946, Arrigoni 2014), *Violamercurii* Orph. ex Halácsy (PI nr. 051514, Reich et al. 2021); syntypes of *Centaurealaureotica* Heldr. ex Halácsy (PI nr. 012698, Reich et al. 2021), *C.tuntasia* Heldr. ex Halácsy (PI nr. 012866, Reich et al. 2021), *Helianthemumjonium* Lacaita & Grosser (PI nr. 051105), *Hieraciumhalacsyi* Heldr. ex Halácsy (PI nr. 019507, Reich et al. 2021), *Poatimoleontis* Heldr. ex Boiss. (PI nr. 029594, Reich et al. 2021), and an isoepitype of *Cirsiumlobelii* Ten. (PI nr. 013207, Del Guacchio et al. 2021).

We also traced original materials of four taxa described by Guadagno himself: BromuserectusHuds.var.stabianus Guadagno (Guadagno 1913), Catapodiumloliaceum(Huds.)Linkf.erectum Guadagno (Guadagno 1913), CerastiumhirsutumTen.var.pumilum Guadagno (Guadagno 1926) and *Rumexpseudoamplexicaulis* Guadagno (Guadagno 1926). In addition, Del Guacchio and al. (2020a) recently described the hybrid Centaurea×cavarae Guadagno ex Del Guacchio, Cennamo & P.Caputo based on a hypothesis that M. Guadagno reported on the label of the holotype PI nr. 034717 about the putative hybrid origin of this plant between *Centaureadeusta* Ten. and *C.montaltensis* (Fiori) Peruzzi (see also De Luca et al. 2023).

The project carried out on the Herbarium Guadagno is part of the ongoing process that was started at the Herbarium of Pisa, currently focused on the digitization of type specimens, historical collections, revised specimens and all the new acquisitions that are being entered in the Herbarium (Astuti et al. 2019; Roma-Marzio et al. 2020; Maccioni et al. 2023). This activity has highlighted the high value of PI-GUAD in terms of geographic and temporal coverage, as well as for the precious material linked to type specimens, and for the historical information linked to documents, iconographies and original drawing found in the Herbarium. The study of PI-GUAD, associated with his card index, will be fundamental for studies concerning the flora of southern Italy and particularly of Campania region. In this framework, although the Herbarium Guadagno represents a small portion of the estimated 400 million specimens deposited across about 3,000 herbaria worldwide, its digitization significantly contributes to the emergence of the so-called open access global metaherbarium, that will be the crucial for guiding the exploration, illumination, and prediction of plant biodiversity change in the Anthropocene, and will facilitate the rapid exploration, synthesis, and dissemination of accurate biodiversity data at unprecedented scales (Davis 2023).

## ﻿Additional information

### Author ORCIDs

Francesco Roma-Marzio https://orcid.org/0000-0002-2184-0311

David Dolci https://orcid.org/0000-0002-3043-9757

Giovanni Astuti https://orcid.org/0000-0001-5790-3516

Lorenzo Peruzzi https://orcid.org/0000-0001-9008-273X

### Data availability

All of the data that support the findings of this study are available in the main text or Supplementary Information.

## Supplementary materials

Supplementary material 1 Supplementary data Data type xlsx https://binary.pensoft.net/file/920550This dataset is made available under the Open Database License (http://opendatacommons.org/licenses/odbl/1.0/). The Open Database License (ODbL) is a license agreement intended to allow users to freely share, modify, and use this Dataset while maintaining this same freedom for others, provided that the original source and author(s) are credited.Francesco Roma-Marzio, Simonetta Maccioni, David Dolci, Giovanni Astuti, Nicoletta Magrini, Federica Pierotti, Roberta Vangelisti, Lucia Amadei, Lorenzo Peruzzi
